# Revision total knee arthroplasty: hybrid vs standard cemented fixation

**DOI:** 10.1186/s10195-018-0494-y

**Published:** 2018-08-17

**Authors:** Jesús Gómez-Vallejo, Jorge Albareda-Albareda, Belén Seral-García, Nieves Blanco-Rubio, Laura Ezquerra-Herrando

**Affiliations:** Department of Orthopedic Surgery, “Lozano Blesa” University Hospital, Joaquina Zamora 4, 4º B, 50018 Saragossa, Spain

**Keywords:** Revision knee arthroplasty, Stem, Cemented fixation, Hybrid fixation

## Abstract

**Introduction:**

Modular systems with stems are necessary for the stability of revision total knee arthroplasty (rTKA), but controversy remains as to the best fixation method: cemented or hybrid (noncemented stem). The aim of this study was to assess the clinical, X-ray, life-quality and survival results obtained with each fixation method.

**Materials and methods:**

During the period 2000–2013, rTKA was performed on 67 patients (29 cemented arthroplasty and 38 hybrid fixation). The average follow-up was 7 years (range 2–15). All patients were evaluated clinically and radiographically using the American Knee Society Score (AKSS), the Western Ontario and McMaster Universities Arthritis Index (WOMAC), and the Short Form Health Survey (SF-36). A survival study was performed via Kaplan–Meier analysis.

**Results:**

There were no differences between the cemented and hybrid fixation groups in the preoperative and postoperative AKSS clinical evaluation indices and the SF-36 health index. However, the WOMAC assessment scale did reveal statistically significant differences between the groups, with a global classification of 64.9 points weighted at 100 (SD 16.8) for cemented fixation versus 78.9 (SD 9.0) for hybrid fixation (*p* = 0.001). The corresponding values for stiffness were 61.6 (SD 12.9) and 80.5 (SD 14.7) (*p* = 0.001), and those for function were 61.3 (SD 19.4) and 78.1 (SD 10.5) (*p* = 0.001). No significant differences between the groups were recorded with respect to the pain score (*p* = 0.4) or the results of the Kaplan–Meier survival analysis.

**Conclusion:**

Although the results were similar for the two groups, hybrid fixation tended to produce better results than cemented fixation. In view of the risk of further loosening, we prefer the more conservative approach, i.e. hybrid fixation.

**Level evidence:**

Level III.

## Introduction

Revision arthroplasty of the knee is one of the great challenges in orthopaedic surgery. The number of prosthesis replacements performed is rising, but the surgical procedure is very demanding: there is very little bone substrate and its architectural quality is poor [1]. In response, modular revision prostheses are viewed as an excellent treatment option [2]. The surgical material used in this type of surgery must be very versatile, as total knee revision arthroplasty poses several very complex problems, especially bone loss and insufficient soft tissue. The use of metal wedges and augmentations to compensate for bone loss has become generally accepted, and morselised or structural bone grafts are also commonly employed to treat bone defects [3].

Constrained prostheses are widely used to overcome the lack of ligaments. However, the constriction system requires the contact surface between bone and prosthesis to withstand a large number of force vectors, and therefore the replacement surface must be cemented [1]. Nevertheless, modular systems with stems are now widely used for strengthening prosthesis stability.

The question then arises: is it actually necessary to cement these central-medullary devices? Cemented stems have been shown to perform well and to be long lasting. However, there are drawbacks, especially the stress shielding that may occur and the difficulty involved in removing the cement if further revision is necessary [4]. Several papers have addressed this question, but few have compared the two systems, and fewer still have employed a life-quality index.

The primary aim of the study reported in the present paper was to assess how the clinical and life-quality outcomes achieved depend on the fixation method employed (hybrid or cemented) in total knee arthroplasty replacement. Secondary outcome measures were survival and X-ray results. The hypothesis of this study was that the hybrid fixation method achieves better clinical and X-ray results than the cemented stem method.

## Materials and methods

The study performed was analytical and retrospective. The study population was obtained by reference to the database maintained at the authors’ institution. The patients selected for study were consecutively operated on from January 2000 to December 2013 for revision total knee arthroplasty (rTKA), including either cemented stem (Natural Knee II, Centerpulse^®^ Warsaw, IN, USA) or hybrid fixation (P.F.C. TC-3 Sigma, DePuy^®^ Raynham, MA, USA). The implant decision was taken by a single surgeon. 97 cases were considered for analysis, imposing the following inclusion criteria: surgery performed at the abovementioned institution in the period January 2000 to December 2013, due to failure of the primary prosthesis; revision surgery performed on only one knee; replacement recommended due to aseptic failure; at least the tibial and femoral components were replaced in this surgery. The exclusion criteria were: septic loosening of the previous implant; prior replacement surgery on the same knee, in any form; failure to provide informed consent to participate in the study. Of the original population, 21 were lost to follow-up, and nine were excluded due to septic loosening of the implant.

Finally, therefore, a sample group of 67 patients was formed, of whom 29 received a cemented arthroplasty and 38 a hybrid fixation arthroplasty. The choice of implant was decided at the surgeon’s discretion, without randomisation.

All these revision surgeries were performed by the same physician (FS). In every case, the bone cement was used without antibiotic and systematic cephazolin was provided.

Low-molecular-weight heparin was given the evening before surgery and then once daily for 1 month. Ambulation began on the second postoperative day with weight bearing as tolerated with canes. Physical therapy included daily range-of-motion exercises, assisted by continuous passive-motion machines. Patients continued physical therapy as outpatients after hospital discharge.

The average follow-up of the revision arthroplasties was 7 years (range 2–15; SD: 3 years). All patients were evaluated prior to surgery.

The epidemiological data collected included age, gender, body mass index and years elapsed since the primary arthroplasty. In addition, the ASA Physical Classification System was applied [5] and bone status was determined according to the Anderson Orthopaedic Research Institute Classification [6].

The following surgery data were recorded: type of procedure, haemorrhage during procedure, duration of surgery and size of insert.

The American Knee Society Score (AKSS) [7] was used to assess the joint, both prior to surgery and at the final postoperative follow up. In the latter examination, the Western Ontario and McMaster Universities Arthritis Index (WOMAC) [8] and the Short Form Health Survey (SF-36) [9] index were also determined. The scores for these two indices were weighted at 100, where 0 is the worst possible result and 100 the best. The AKSS, WOMAC and SF-36 indices were taken as the primary outcome measures.

**Fig. 1:**
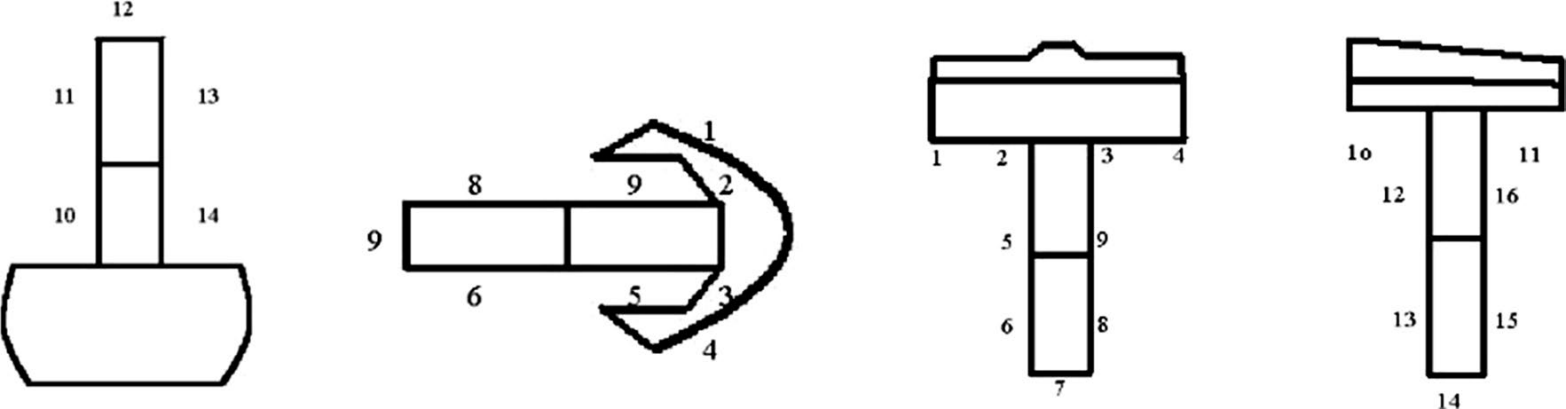
Areas in the Fehring modified AKSS X-ray evaluation

The modified AKSS proposed by Fehring [10] was used to perform the evaluation of the final X-ray image obtained. To facilitate analysis of the stem, the implant was divided for study into specific areas: 14 for the femur and 16 for the tibia (see Fig. 1). The radiolucency lines were measured in millimetres in each area, and are shown according to the implant performed. For the femur, an implant was considered stable if the total thickness of these radiolucency lines was no more than 8 mm; from 9 to 19 mm, monitoring was required, and 20 mm or more reflected the existence of a loose implant. On the tibia, the corresponding values were ≤ 9, 10–22 and ≥ 23 mm.

An implant was considered to have failed when the patient subsequently required a revision of any of the prosthetic components or was on the surgical waiting list for the same reason. In our study, six of the implants were classed as loose: four were cemented and two were hybrid fixations.

With respect to the primary outcome measures, the Student *t* test was used to compare the quantitative variables that followed a normal distribution with the dichotomous variables (hybrid vs. cemented). The Kolmogorov–Smirnov test was applied to determine whether quantitative variables were normally distributed. The Pearson correlation test was used to verify the relationship between two quantitative variables. In all cases, 95% confidence intervals were calculated. For the secondary outcome measures, the chi-square test was used to compare the qualitative variables. The survival study was performed by Kaplan–Meier analysis, with statistical significance assumed at *p* < 0.05.

## Results

The preoperative and postoperative AKSS clinical evaluation indices and the SF-36 health index revealed no differences by cementation type (Table 1).

**Table 1:** AKSS and SF-36 results for the cemented (c) and hybrid (h) arthroplasties

	Type	*N*	Mean	Standard deviation
AKSS function (*p* = 0.12)	c	29	77.80	11.87
	h	38	72.26	16.17
AKSS (*p* = 0.82)	c	29	70.65	18.04
	h	38	71.65	19.08
SF physical (*p* = 0.32)	c	29	60.99	13.42
	h	38	64.70	16.13
SF mental (*p* = 0.14)	c	29	66.19	11.51
	h	38	70.83	13.50
SF-36 (*p* = 0.11)	c	29	64.32	13.28
	h	38	69.43	12.65

The presence of pain, according to the AKSS scale, also did not differ statistically significantly between the cemented and hybrid fixation groups. In the hybrid-fixation group, there were only three cases (8%) of pain at the tip of the shaft in the tibia, and the number of such cases in the cemented group was not statistically significant either.

The average postoperative flexion was 89° (SD 17) among the cemented arthroplasties and 90° (SD 20) among the cases of hybrid fixation. For extension, a statistically significant difference was found (3°, SD 6 vs 1°, SD 3) favouring cemented fixation (*p* = 0.04). Joint balance was not statistically significant between the groups (88.2°, SD 17.3 in the cemented-fixation group and 86.8°, SD 23.4, in the hybrid-arthroplasty group; *p* = 0.74).

The WOMAC assessment scale did present statistically significant differences in global classification (64.9 points weighted at 100 for the cemented arthroplasties, SD 16.8; and 78.9, SD 9.0, for the hybrid ones; *p* = 0.001: 95% CI 7.62–20.38), stiffness (61.6, SD 12.9; and 80.5, SD 14.7; *p* = 0.001: 95% CI 12.03–25.77) and function (61.3, SD 19.4, and 78.1, SD 10.5 (*p* = 0.001: 95% CI 9.42–24.18). However, no such differences between the groups were observed for the pain score (*p* = 0.4).

**Fig. 2:**
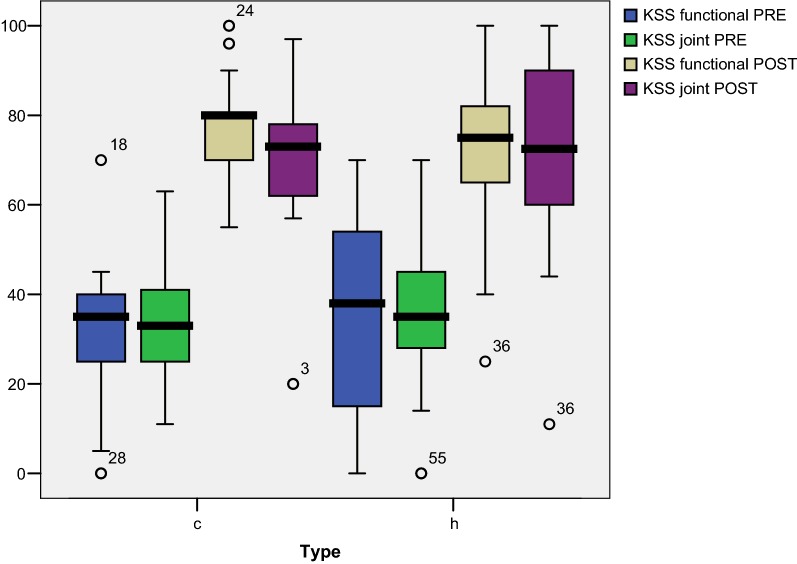
Group comparison [cemented* (c)* and hybrid* (h)*] of the pre- to postoperative variation in the AKSS index

No differences between the groups were found in the pre- to postoperative variations of the AKSS index (*p* = 0.63 joint scoring and *p* = 0.09 functional scoring) and joint balance (*p* = 0.79). We conclude, therefore, that the improvement achieved was the same for both types of prosthesis fixation (Fig. 2).

No statistically significant differences between the treatment groups were found for age, gender, ASA or body mass index (Table 2).

**Table 2:** BMI and age results for cemented (c) and hybrid (h) arthroplasties

	Type	*N*	Mean	Standard deviation
BMI	c	29	31.1138	3.69040
	h	38	31.2158	4.43651
Age	c	29	79.7241	4.37441
	h	38	78.3684	4.43220

Similarly, there were no significant differences between the groups in the operation variables. The surgery time was practically the same (143 min for both cemented and hybrid fixations, SD 23.5 and 23.7, respectively). The amount of bleeding was slightly higher in the hybrid fixation arthroplasties, where the tourniquet was removed before closing the joint (308 cc, SD 137 vs 282 cc, SD 211 in the cemented fixations). There were no differences between the groups regarding the use of bone grafts or augmentations, or in the Anderson classification (mode 2A for the tibia and 2B for the femur). For the cemented arthroplasties, the mode value for the length of the stem in the femur was 125 mm (80%), and for the diameter it was 10.5 mm. The most commonly used types of stem in the tibia measured 60 mm and were modular and conical. In the hybrid fixation group, the mode value for the length of the femur component was 125 mm and for the diameter it was 14 mm, while the respective values for the tibia were 115 and 12 mm.

The patients were followed up at 4 weeks after the operation and again after 2, 4, 6 and 12 months. Subsequent follow-ups were yearly, and anteroposterior and lateral X-rays were taken on each occasion.

Assessment of the radiological stability of the implants did not reveal any significant variations, with a mean value of 2 (range 0–19) for the cemented femurs and 3 (range 0–12) for the hybrid fixations (*p* = 0.22). According to the Fehring classification, the mean value was 4 (0–12) for the cemented tibias and 3 (0–10) for the hybrid fixations (*p* = 0.15). Among the cemented fixations, four implants had possibly loosened in the tibia and two in the femur. No case was defined as migrated. Among the hybrid fixations, there was one possibly loose implant in the tibia and four in the femur. Here, too, there were no migrations.

Six of the arthroplasties were classed as failed—three due to late-onset infection, one because of femoral stem breakage caused by fatigue after 6 years of evolution in a cemented prosthesis, and two due to instability.

**Fig. 3:**
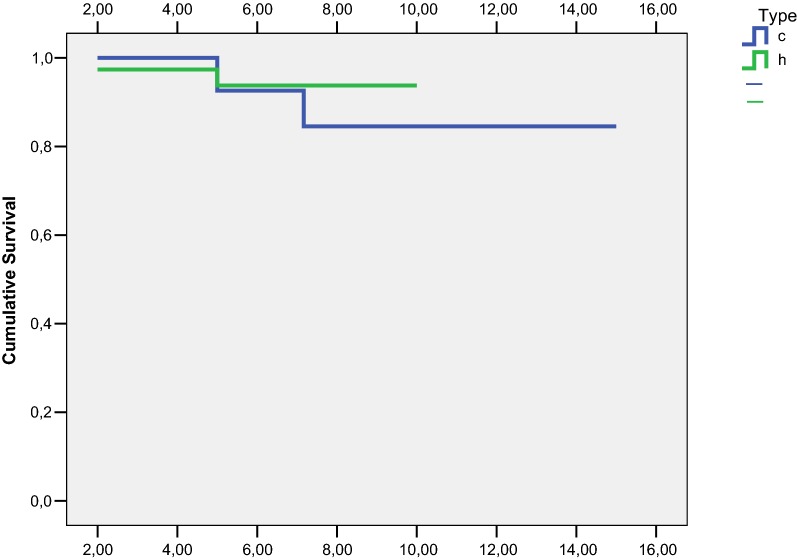
Kaplan–Meier survival curves for cemented (*c*) and hybrid (*h*) arthroplasties

Regarding the survival of the implants, using any re-revision as the endpoint, no statistically significant difference between the groups was found. Over time, both curves were similar: at 2 years (100% in the cemented-fixation group and 97% (CI 93–101) in the hybrid-implant group), at 5 years (93%, CI 83–103 and 94%, CI 85–101) and at 10 years (84%, CI 70–98 and 94%, CI 86–102). In the cemented cases, the corresponding values were notably lower, but the difference did not reach statistical significance (*p* = 0.61) (Fig. 3).

## Discussion

In studies of revision arthroplasty, the type of fixation that should be employed is a question that has attracted much attention. However, very few papers have compared the two main types of fixation: cemented and hybrid. The main conclusion reached in the present study is that the two systems achieve similar clinical results and survival times.

The biomechanics of these implants have been studied in numerous experimental models. Thus, Completo et al. [11, 12] studied the fixation of implants in vitro in revision implant surgery, analysing the behaviour of the proximal tibia using a synthetic model. Those authors reported that load distribution in the tibia was better with a long, noncemented stem. On the other hand, another study conducted in the same year to examine the behaviour of implants in tibia bone defects reached the conclusion that both types of fixation performed similarly in cavitary defects, although the cemented implant seemed to be the better option for large defects treated with structural allografts. However, in a clinical study of large-scale osteolysis, the authors recommended hybrid fixation, although this decision should be taken according to the bone quality observed (evidence level III) [13].

Jazrawi et al. [14] conducted a study, based on an examination of 12 cadavers, of the pressure vectors presented by cemented and hybrid stems, and concluded that with cemented stems there is less micromovement of the implant but increased stress shielding on the proximal tibia. Therefore, for an impacted stem to achieve a fixation as stable as that provided by a cemented implant, it must be larger (specifically, 75–150 mm).

Skawara et al. [15] studied cadavers subjected to cyclical loads. Radiostereometric analysis showed that the load distribution was more regular in hybrid fixations and failed less often than in cemented implants. Accordingly, those authors recommended the former approach, although it should be noted that the fixations they studied were primary implants. In summary, the findings of those studies are inconclusive, and both methods seem to obtain similar results.

Few studies have reported results obtained from implants with stems for knee arthroplasty revision, and most of those studies are retrospective [16].

Some papers have discussed cemented and hybrid revision arthroplasties, but without performing a direct comparison of the results obtained [17, 18]. Thus, Kim et al. analysed surgical interventions—cemented or hybrid fixations—performed on 114 knees and calculated the WOMAC score before and after surgery (15 and 65.5, respectively, vs the global postoperative score of 72.3 obtained in our analysis), but without differentiating between types of fixation.

In our own study, there were no statistically significant differences regarding the AKSS and the SF-36 indices, although the results were better with hybrid fixation. There were differences between the groups in the WOMAC scores for function and stiffness, but not for pain. There were no differences regarding the improvement gained according to the AKSS and the measurements of joint flexion, extension and balance (before and after surgery). These findings suggest that both systems are appropriate and equally effective. This point highlights one of the weak areas of our study: we did not have access to preoperative WOMAC and SF-36 scores because this procedure was not initiated in our department until after the study began.

Cemented fixation has probably been less extensively studied than the hybrid approach, but more research in this area is now being undertaken [19, 20]. In this respect, the poorest results are those reported by Whaley et al. [21], with an AKSS function score of 56.9 (from a basal value of 16) and a knee score of 51 (from a baseline of 48), and the best ones are those noted by Mabry et al. [22], with an AKSS function score of 85 and a knee score of 91.

By contrast, many papers have praised the virtues of hybrid fixation [1, 23, 24], reporting good-to-excellent results and recommending this surgical method. The results obtained for functional AKSS range from 55 in the worst series to 82 in the best, and from 79 to 85 for joints [2, 25–29].

Following Vince and Longe [25], many advocates of cemented fixation have criticised the use of constrained implants with hybrid fixation for revision knee arthroplasty. However, the failures observed by these authors occurred in septic revisions, where there was more severe structural damage and outcomes were inevitably worse. Other, well-designed studies [17, 30–32] recommend the use of constrained prostheses such as TC-III, reporting very good results from this approach, and they reserve the cemented-stem method for hinged prostheses presenting considerable loss of bone structure.

Another factor that should be taken into account is that the range of variables considered in reports of hybrid revisions is much narrower than in those focusing on cemented fixations, which would make the former results appear more predictable.

According to our literature review, few studies have been undertaken to compare X-ray images by type of prosthesis fixation (cemented or hybrid). Among this limited body of research, Peters et al. [18] observed no differences according to the type of fixation, although there appeared to be more lucency around the metaphyseal area in the hybrid-fixation implants (performed using a different technique to that examined here), which was plausibly explained by the nonimpaction of cement in that area. Fehring et al. [10] examined a series of hybrid-fixation arthroplasties and reported that 71% were stable, 19% were possibly loose and 10% had migrated—values that were significantly different from those obtained for a corresponding cemented series, with 93% stable and 7% possibly loosened. However, as mentioned above, that study concerned the comparison of metaphyseal stem X-rays and did not address the question of survival, nor mention any clinical scale. Reported radiolucency rates are similar for both types of fixation, suggesting that the type of fixation employed does not affect the X-ray results obtained [33–36].

Our study revealed no significant differences in survival between the two types of fixation. Although there was a difference of 10% between the respective survival rates at 10 years, this difference was only 1% at 5 years.

In our literature review, we also considered the longevity of the implants, taking into account only those results obtained by Kaplan–Meier estimation or similar, without regard for the percentage of failures. In this respect, cemented arthroplasties were again the least-commonly studied; only two papers addressed this question, reporting survival rates of 97% at 5 years and 89–94% at 10 years (in contrast to the 84% found in our series) [18, 19]. For hybrid-fixation arthroplasties, the results were similar. The series were somewhat longer in time than those of the cemented fixations, and the values obtained ranged from 92 to 95% at 5 years (93.7% in our series of hybrid fixations) to 83–93.5% at 10 years (94% in our series) [2, 18, 28, 29, 37, 38]. Sheng et al. [31] recorded greater longevity for cemented than for completely noncemented fixations. A final consideration is that the research in this field was basically carried out between 2000 and 2012, and so the outcome over the last decade remains to be established.

This study has certain limitations. The number of subjects required was not determined a priori. Moreover, the study sample is relatively small (although this is also the case in most of the studies reviewed). In addition, the study groups were not randomised, and this reduces the statistical power obtained.

Taking into consideration the data previously reported, together with the findings presented here, we conclude that there are no great differences, in practice, between the two types of fixation. Cemented and hybrid fixations both constitute reproducible, safe procedures. Nevertheless, in view of the possible need for further revisions, and taking into account the complexity of the removal of cement, our choice is to use hybrid stems.
